# Subconscious vibrotactile stimulation improves mobility and balance in patients with bilateral vestibulopathy: adherence over 2 years

**DOI:** 10.3389/fneur.2024.1491195

**Published:** 2024-10-08

**Authors:** Herman Kingma, Dan Dupont Hougaard, Raymond van de Berg

**Affiliations:** ^1^Department of Otorhinolaryngology and Head and Neck Surgery, School for Mental Health and Neuroscience, Maastricht University Medical Center, Maastricht, Netherlands; ^2^Balance and Dizziness Centre, Department of Otorhinolaryngology, Head and Neck Surgery and Audiology, Aalborg University Hospital, Aalborg, Denmark; ^3^Department of Clinical Medicine, Aalborg University, Aalborg, Denmark

**Keywords:** bilateral vestibular loss, vibrotactile stimulation, balance, vestibular prothesis, rehabilitation, balance belt, quality of life, mobility

## Abstract

**Objective:**

To investigate the effect of daily use of subconscious vibrotactile stimulation in bilateral vestibulopathy (BVP) patients, who judged the effect of vestibular rehabilitation as insufficient.

**Methods:**

BVP patients were asked to wear a subconscious vibrotactile stimulation belt for 2 h. Patients who reported benefit after 2 h of use, were instructed to wear a subconscious vibrotactile stimulation belt in daily life, for up to more than 2 years. Follow-up consultations (mostly by telephone calls) were scheduled after 2 weeks, 2 months, 1 year, and 2 years of use. During these consultations, adherence and the self-reported overall Balance and Mobility Score (BMS) were evaluated.

**Results:**

One hundred twenty-one BVP patients were included. Regarding adherence, 74% of patients (*n* = 89) wanted to proceed with daily use at home after 2 h of try out. Of these patients, 90% (*n* = 80) was still wearing the belt daily after 2 months, and at least 81% (*n* = 72) after 1 year and 73% (*n* = 65) after 2 years. It should be noted that lack of adherence after 1 and 2 years resulted from a loss to follow-up. All patients responding to telephone consultations in the 2 years follow up were wearing a subconscious vibrotactile stimulation belt daily. The median BMS score significantly improved within 2 h of use, from 4 to 6 points (*p* < 0.0001). Compared to baseline, the median BMS score significantly improved with >=3 points after 2 weeks, 2 months, 1 year, and 2 years of daily use (*p* < 0.0001). Long-term adherence was high in patients who experienced an increase of two or more points on the BMS, after 2 weeks of daily use.

**Conclusion:**

The Subconscious vibrotactile stimulation improves self-reported balance and mobility in a subgroup of motivated BVP patients in which vestibular rehabilitation is insufficient.

## Introduction

Bilateral vestibulopathy (BVP) is a chronic vestibular syndrome. The most frequent symptom of BVP is imbalance (91.4%), followed by chronic dizziness (57.7%) and oscillopsia (50.1%). In this syndrome, the gain of the vestibular ocular reflex (VOR) is bilaterally reduced in the video-head impulse and/or caloric test and/or torsion swing test (1).

Unfortunately, the worldwide prevalence of BVP is still not very precisely documented. This is (partially) related to two important aspects: (1) the varying definitions and vestibular assessments in literature (2, 3), and (2) misdiagnosis due to the absence of vertigo (39). Vertigo is often considered an obligatory symptom in vestibular disorders, but it is not always present in BVP patients (2). Therefore, non-experts (11) might easily overlook a vestibular origin of imbalance (4, 5). This is unfortunate, since more than nine percent of elderly with dizziness, referred to a falls clinic, could be diagnosed with BVP (6, 37).

Fact is that in the Netherlands, about 30% of the independently living elderly over 65 years old fall at least one time per year, with injuries in 40–60% of the falls associated with injuries and high costs. More than 6,200 elderly died after a fall in 2022 in the Netherlands (7). Therefore, imbalance and falls are a major socio-economic problem.

Although BVP is mainly reported in the elderly, it is also observed in infants. Especially children with severe sensorineural hearing loss eligible for cochlear implantation, are at risk (9, 10, 38). Major symptoms in these infants are delayed motoric development and imbalance, but the impact of BVP reaches much further and affects many aspects of child development (8–10). It could therefore be hypothesized that infants might also benefit from vestibular therapy.

Regarding vestibular therapy for BVP, many options are currently available in clinic or research settings. The major current options are:

1. Vestibular Rehabilitation. Vestibular rehabilitation improves balance and mobility in elderly, strengthens muscles and optimizes behavioral adaptation mechanisms (12, 13, 34). However, there is doubt about the effectiveness of vestibular rehabilitation to prevent falls and to improve fall strategies to avoid serious injuries (14).
2. Specific fall prevention training. Fall training or Perturbation Based Training is suggested and under study as a specific tool to prevent falls and associated injuries in elderly (14).
3. Galvanic Stimulation. Continuous noisy subconscious and sub-threshold galvanic (electric) stimulation of the labyrinth might improve balance. The technique is non-invasive because the electrodes are placed on the skin. It is hypothesized that the noisy stimulation adds up to the physiological movement signal and this sum signal exceeds the vestibular threshold for motion and tilt, leading to improved perception and balance (16, 17).
4. Visual or vibrotactile feedback to optimize vestibular rehabilitation: To see or feel the body motion can support balance training (15, 36).
5. Vibrotactile feedback for daily use. Subconscious ambulatory vibrotactile stimulation can be achieved by a belt worn around the waist. It is used then during standing and activity during the day, holding 10 tactors (mini vibrators) and a tilt and motion sensor. The belt constantly measures trunk tilt and acceleration during activities. A microprocessor codes this information into a specific vibration pattern around the waist. These vibrations facilitate somatosensory detection of trunk movement and tilt, to substitute for severe BVP. It improves self-reported balance and mobility and is superior to placebo (18–20). However, when vibrotactile feedback is used in daily life to consciously control posture, as in rehab, it becomes a primary task disturbing normal function and prevents subjects from executing normal other tasks (37). This is what we observed in many pilot studies. Therefore, patients need to be instructed not to pay attention to the vibration and to move and walk around as much as possible as they did before the vestibular problems started: this application is named subsconscious vibrotactile stimulation.
6. Vestibular Implant (VI). Motion sensors capture head movements and a microprocessor codes these head movements into electrical pulses. These pulses are transferred to the vestibular nerves by surgically implanted electrodes in the semicircular canals or otolith organs (32). The first multichannel vestibular implant in a human in the world was implanted in 2012 by a research team from Geneva and Maastricht (21). In the last decade it was demonstrated by several research groups that a vestibular implant can improve balance, reduce oscillopsia and improve posture and gait (21–33). The vestibular implant is not (yet) clinically available. However, results show that it might become a clinically useful device in the near future (32).

The objective of this study was to investigate the effects of daily use of subconscious vibrotactile stimulation as a non-invasive device in BVP patients.

## Methods

### Study design

In this study, BVP patients were asked to wear subconscious vibrotactile stimulation for 2 h. After 2 h of use, patients could decide whether they wanted to continue using subconscious vibrotactile stimulation. Patients who wanted to continue, were instructed to wear the subconscious vibrotactile stimulation in daily life, for up to more than 2 years. Follow-up consultations were scheduled after 2 weeks, 2 months, 1 year, and 2 years of use. During these consultations, adherence and self-reported overall Balance and Mobility Score (BMS) were investigated. Due to the COVID-pandemic, follow-up consultations were mainly performed by telephone and BMS was chosen as the only outcome parameter (20).

### Patient inclusion

Bilateral Vestibular Pathology patients were recruited from a tertiary referral center (Maastricht University Medical Center). Patients who considered vestibular rehabilitation insufficient as a treatment were selected.

Inclusion criteria comprised (1):

1. Severe imbalance with a fear to fall and/or actual falls.
2. At least 1 month after finalizing vestibular rehabilitation, which (according to the patient) insufficiently improved quality of life.
3. Self-reported overall Balance and Mobility Score (BMS) ≤ 5 (test range 0–10).
4. Laboratory test results indicative of BVP (1):

a. Sum of bithermal maximum peak slow phase velocity < 6°/s of the caloric nystagmus on each side, in the caloric test with water (30 and 44°C) and
b. Vestibulo-ocular reflex gain of < 10% on rotatory chair test (sinusoidal stimulus, 0.1 Hz, peak velocity 50°/s) and/or
c. Bilateral pathological head impulse test for horizontal semicircular canals (vestibulo-ocular reflex gains < 0.6).

5. Quality of health score (QHS) as derived from the SF-36 < 40%.
6. Able to walk (walking stick or walker allowed).

Patients with neurological, psychiatric, or orthopedic disorders were excluded, as well as patients with reduced proprioceptive sensitivity and impaired vision despite correction.

### The vibrotactile stimulation belt

**Figure 1:**
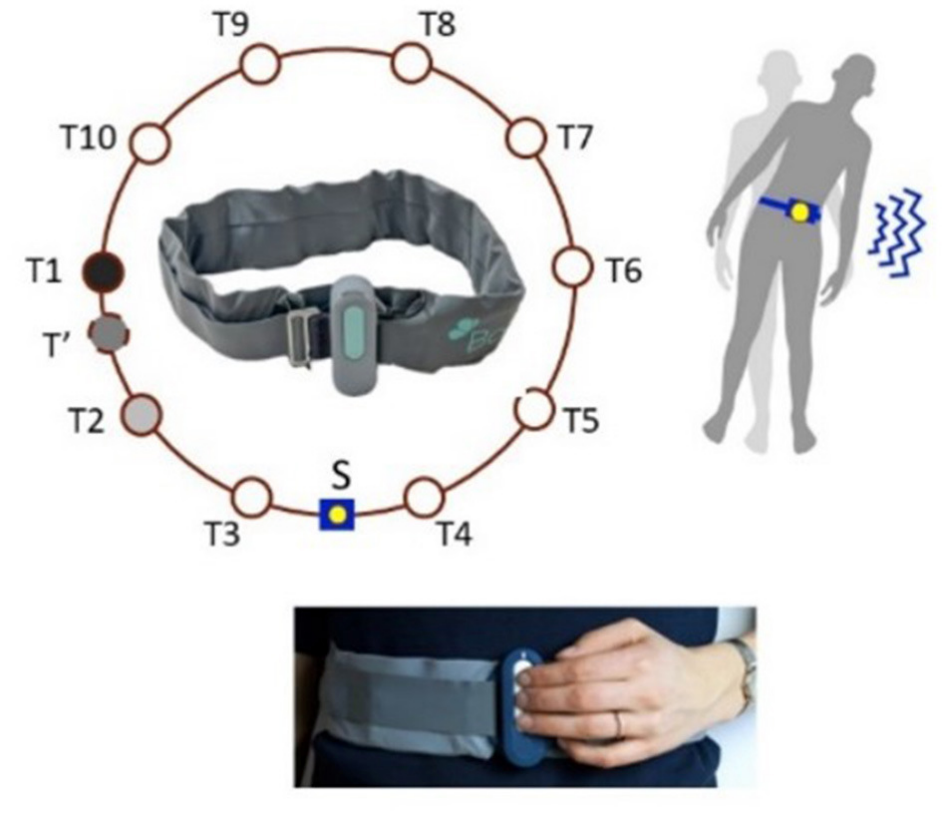
Position of 10 tactors, sensor and control buttons of the vibrotactile stimulation belt. S, sensor and microprocessor; T1–T10, tactors.

The vibrotactile stimulation belt was developed and studied as a non-invasive prothesis to improve balance and mobility in BVP patients (18–20). Details regarding this technique can be found in previously published articles (18–20). In summary, the vibrotactile stimulation belt provides a vibration pattern around the waist, using 10 tactors that are built in the belt. The tactors are placed every 36 degrees, equally distributed over the belt. The tactors are activated via the microprocessor using a transfer function based on the trunk movement and tilt sensed by the 6DOF sensor. By simultaneously activating two tactors (for example T1, T2) with different intensities (linear interpolation), virtually every part of the (for example T') can be stimulated (Figure 1). For this study we used a programmable vibrotactile stimulation belt, the BalanceBelt, designed and produced by Elitac Wearables Company, Utrecht, The Netherlands.

### Outcome measures: adherence and BMS

Adherence was defined as the percentage of patients that kept using the vibrotactile stimulation belt in daily life setting, after deciding to wear it at home. Patients who did not want to try the vibrotactile stimulation belt at home, were not included in the adherence analysis. To not overestimate the effect of the vibrotactile stimulation, missing data (i.e., loss to follow-up) were considered as “lack of adherence.”

The BMS scores the balance and mobility on a scale from 0 to 10. A score of 0 is equivalent to the inability to stand without support; a score of 10 refers to the balance and mobility that an individual patient had before the imbalance and limitation in mobility started [the patient's personal best, see (20)]. Patients were asked during the subsequent consults to score their BMS. Most BMS assessments were done by telephone due to the COVID-pandemic restrictions.

### First consultation and instructions

During the first consultation, patients were able to wear the belt in the hospital for 2 h, as a first try out. They were explicitly instructed to not pay any attention to the vibrotactile stimulation, in contrast to the use of other vibratory training devices used for vestibular rehabilitation (15, 35, 36). They were asked to challenge their balance: they were instructed to be careful, but without support move and walk much as possible. Activities included for example: climbing stairs, riding the elevators, making head- or body turns, or moving their head while walking. After the 2 h of use, patients were asked about their initial impression, and if they wanted to continue the use of the belt at home for 2 weeks (and more). Additionally, the BMS was scored before and after the 2 h of use.

Patients who wanted to wear the belt at home were instructed to use it during daily activities. The belt was not worn during the night, to allow the battery to be recharged.

### Follow-up consultations

Follow-up consultations were scheduled after 2 weeks, 2 months, 1 year, and 2 years of use. Originally the aim was to have in-person follow-up consultations at the clinic, but due to the COVID-19 pandemic, the majority of consultations were done by means of telephone calls. At these consultations, adherence and BMS were investigated.

Furthermore, patients were again explicitly instructed to not pay any attention to the vibrotactile stimulation, and to challenge their balance: they were instructed to be careful, but without support as much as possible. This could include e.g., trying to ride a bike again under safe conditions.

### Statistics

Statistics was performed using Excel, Microsoft^®^ Excel^®^ for Microsoft 365 MSO (Version 2404 Build 16.0.17531.20140) and SPSS (IBM SPSS Statistics for Windows, Version 29.0.2.0 Armonk, NY: IBM Corp). Statistical tests included the Wilcoxon-Signed Rank test, *T*-Test, paired *T*-Test.

### Ethical considerations and approval

The procedures in this investigation were in accordance with the legislation and ethical standards on human experimentation in the Netherlands and in accordance with the Declaration of Helsinki (amended version 2013). The study protocol was approved as non-WMO (Wet Medisch-Wetenschappelijk Onderzoek) research by the medical ethical testing committee Maastricht University Medical Center (METC 2024-0297). This study followed the guidelines outlined by Dutch legislation.

## Results

### Patient characteristics

**Figure 2:**
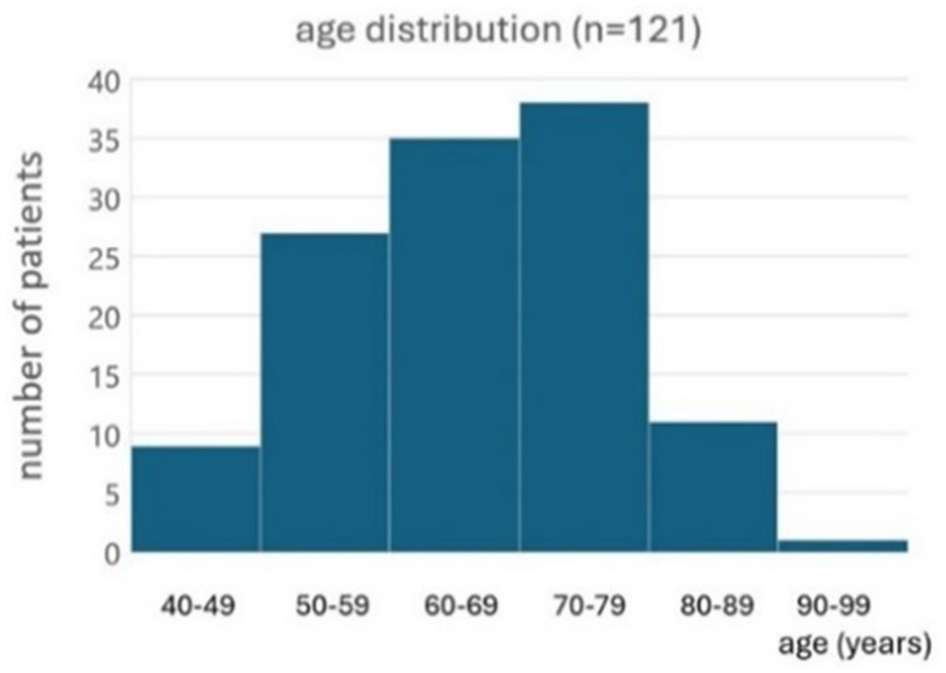
Histogram of the age distribution of patients included to evaluate the subconscious vibrotactile stimulation as a treatment option (*n* = 121).

One hundred twenty-one BVP patients tried the subconscious vibrotactile stimulation for 2 h during the first consultation. Ages ranged from 24 to 94 years old (Figure 2). Etiologies included: idiopathic (*n* = 78), ototoxicity (*n* = 22), genetic/DFNA9 (*n* = 9), bilateral Menière's disease (*n* = 7), and auto-immune (*n* = 5).

### Adherence and balance and mobility score during the first 2 months

**Figure 3:**
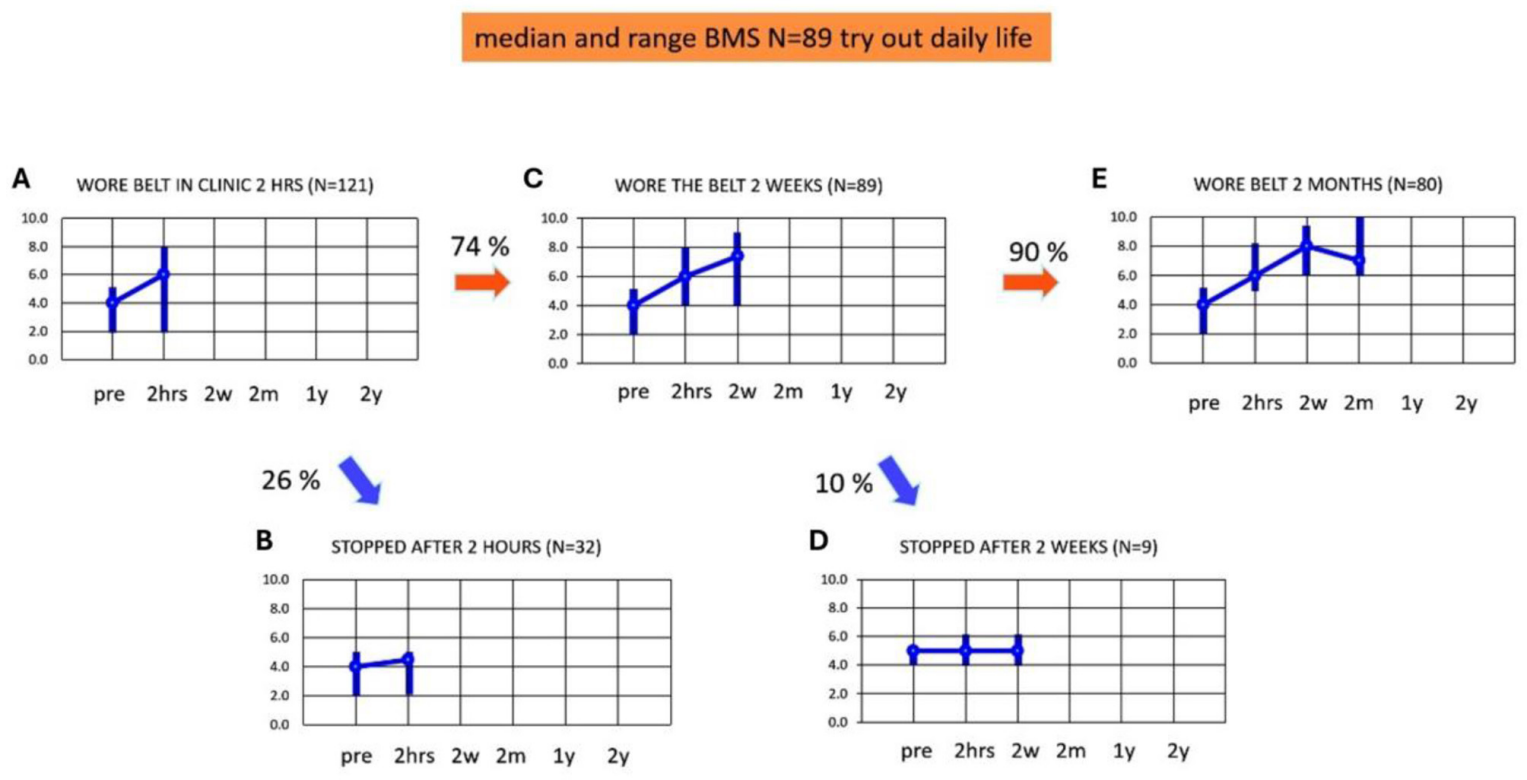
**(A–E)** Adherence and balance and mobility score (y-axis, median and ranges). Results were obtained as a function of time (x-axis) at intake (pre) and after wearing the subconscious vibrotactile stimulation belt for 2 h, 2 weeks, and 2 months. BMS, Balance and Mobility Score; pre, before wearing the belt (baseline); 2 hrs, 2 hours; 2w, 2 weeks; 2 m, 2 months; 1 y, 1 year; 2 y, 2 years. **(A)** BMS at intake (pre) and after 2 h of wearing the belt (*n* = 121). **(B)** BMS of patients excluded from this study as they decided to stop wearing the belt after 2 h of use (*n* = 32). **(C)** BMS of patients who decided after 2 h of use, to continue wearing the belt for 2 weeks (*n* = 89). **(D)** BMS of patients who decided after 2 weeks of use, to stop wearing the belt (*n* = 9). **(E)** BMS of patients who decided after 2 weeks of use, to continue wearing the belt (*n* = 80).

Figures 3A–E present the adherence and median BMS scores in the first 2 months of using subconscious vibrotactile stimulation. Median baseline BMS at intake was 4.0 and BMS ranged from 2 to 5 (*n* = 121). After 2 h use of the Subconscious vibrotactile stimulation in the hospital, the median BMS increased to 6.0 (range 2–8, *n* = 121, Figure 3A). However, 26% of these patients (*n* = 32) did not experience sufficient benefit upon balance and mobility and were excluded as they decided not to enter the study (Figure 3B); their median BMS increased from 4 (range 2–6) to 4.5 (range 2–5, *n* = 9), which was not significant (Wilcoxon-Signed Rank Test, *p* > 0.05 NS). Their individual increase in BMS was always ≤ 1.

Eighty-nine of 121 patients (74%) decided after 2 h use to continue wearing the belt for 2 weeks at home. These patients were considered “2-h responders” and were included in the longitudinal study (*n* = 89, Figure 3C). They had a median BMS of 4 (range 2–5) at baseline, which significantly increased after 2 h to a median BMS of 6 (range 4–8; Wilcoxon-Signed Rank Test, *p* < 0.05). Their BMS significantly increased further after 2 weeks to a median BMS of 7 (range 4–9, Wilcoxon-Signed Rank Test, *p* < 0.05).

Fifty-eight of the 89 included patients experienced benefit after 2 h use. However, 31 (35%) patients did not experience a real benefit after 2 h: their individual increase of BMS was ≤ 1 compared to baseline. This increase was not significant (Wilcoxon-Signed Rank test, *p* > 0.05 NS). These 31 patients nevertheless choose to try the belt at home for another 2 weeks. In nine out of these 31 patients (10%, Figure 3D) the median BMS after 2 weeks remained unchanged: 5 (range 4–6). This implied that they did not experience sufficient benefit after 2 weeks (Wilcoxon-Signed Rank Test, *p* > 0.05 NS). These patients decided to stop wearing subconscious vibrotactile stimulation for a longer period of time. Four out of these nine patients found the vibratory stimulation disturbing. However, the remaining 22 patients out of the 31 patients who did not experience a benefit after 2 h of use, now experienced a benefit of 2 points or more in BMS after 2 weeks use. This implied that 80 patients decided to continue wearing subconscious vibrotactile stimulation for more than 2 weeks. These patients were considered “2-week-responders.” The median BMS score in all 2-weeks responders (*n* = 80, Figure 3E) was 4 (range 2–5) at baseline, which increased significantly by 2 points to 6 (range 5–8) after 2 h (Wilcoxon-Signed Rank Test, *p* < 0.05). Median BMS increased significantly further by 3 points to 8 (range 6–9, *n* = 80) after 2 weeks of using subconscious vibrotactile stimulation (Figure 3E, Wilcoxon-Signed Rank Test, *p* < 0.05).

After 2 months, the median BMS score decreased from 8 (range 6–9) after 2 weeks, to 7 (range 6–10) in these 80 patients (Figure 3E). This decrease was significant (Wilcoxon-Signed Ranked Test, *p* > 0.034). All patients experienced an increase of BMS > 2 after 2 months of using subconscious vibrotactile stimulation.

### Adherence and BMS from 2 months to 2 years

**Figure 4:**
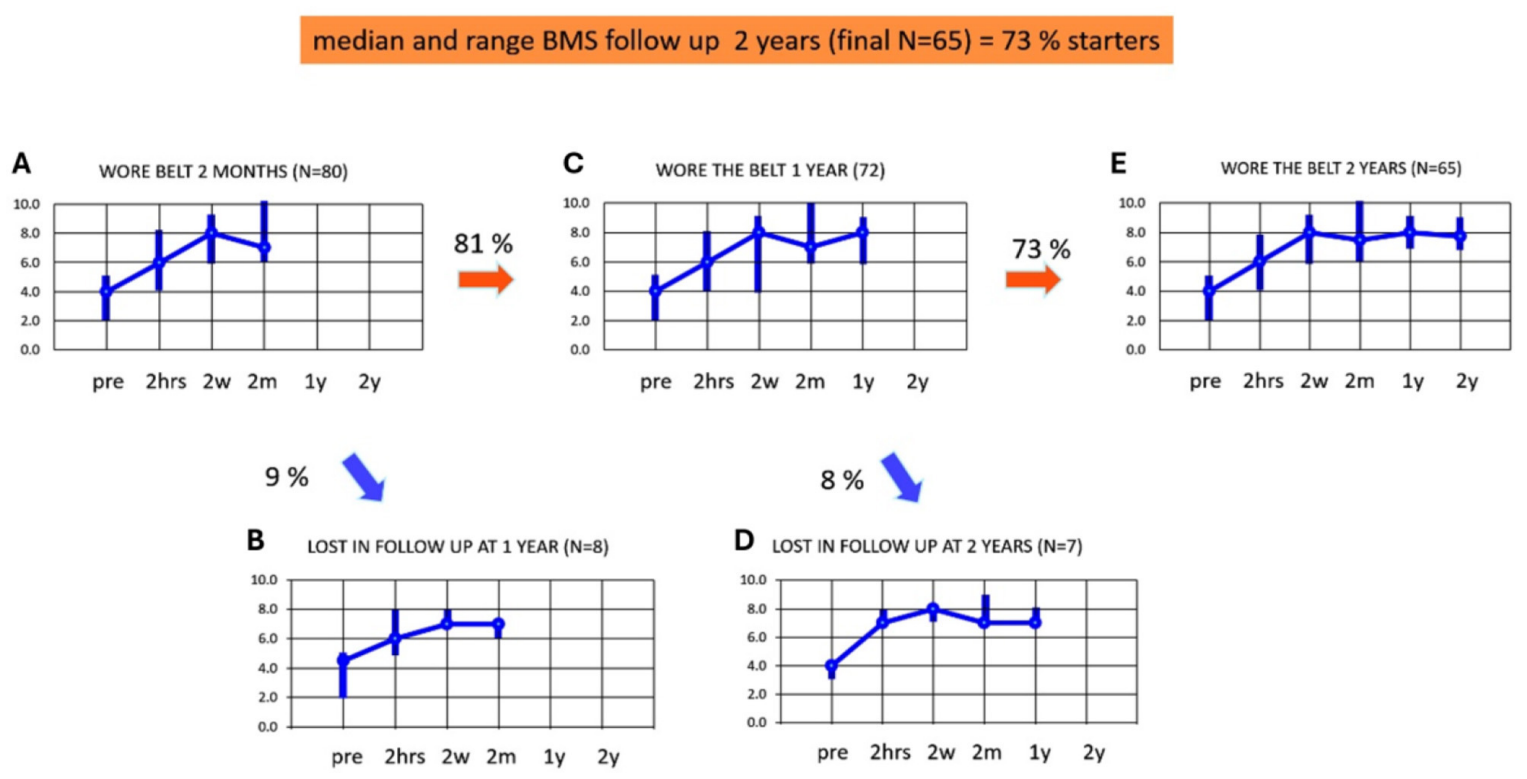
**(A–E)** Adherence and balance and mobility score (y-axis, median and ranges) in patients who decided to continue using subconscious vibrotactile stimulation as a function of time (x-axis), after 2 weeks of use (2-weeks responders). Results were obtained at intake (pre) and after wearing subconscious vibrotactile stimulation for 2 h, 2 weeks, 2 months, 1 year, and 2 years. Pre, before wearing the subconscious vibrotactile stimulation belt (baseline); 2 hrs, 2 hours; 2 w, 2 weeks; 2 m, 2 months; 1 y, 1 year, 2 y, 2 years. **(A)** BMS in the 80 2-weeks responders. **(B)** BMS of the nine patients that were lost in follow-up after 1 year. **(C)** BMS of 72 patients still wearing the belt after 1 year of follow-up. **(D)** BMS of seven patients lost in follow-up after 2 years. **(E)** BMS of 65 patients still wearing the belt after 2 years of follow-up. This involved 65 out of 89 patients included in this study (73%).

Figures 4A–E illustrates the adherence and median BMS scores up to 2 years of using subconscious vibrotactile stimulation, in the 2-weeks responders (*n* = 80). Figure 4A shows the median and range of BMS in these 2-week-responders during the assessment over 2 months (similar to Figure 3E). Eight patients were lost to follow-up in the first year (Figure 4B). The median BMS in the remaining 72 patients was 7 after 2 months (range 6–10, Figure 4C) and 8 after 1 year (range 6–9). The BMS after 2 months and 1 year did not significantly change (Wilcoxon-Signed Rank Test, *p* > 0.05).

Seven patients were lost to follow-up in the 2nd year (Figure 4D). The median BMS in the remaining 65 patients was 8 (range 7–9, Figure 4E) after 1 and also after 2 years. The BMS after 2 months, 1 year, and 2 years did not significantly change (Wilcoxon-Signed Rank Test, *p* > 0.05) in this group.

It should be noted that all patients *responding* during follow-up between 2 months and 2 years, still wore the subconscious vibrotactile stimulation belt. The decrease in adherence between 2 months and 2 years could be fully attributed to the 15 patients who were lost in follow-up. Reasons for loss to follow-up included: deceased (*n* = 6), and not responding to telephone calls (*n* = 9). The median BMS scores of the patients lost to follow-up did not seem to differ from the patients who did respond to the follow-up consultation (see Figure 4: samples were too small to test for significance). The group of 65 patients who were still in follow-up after 2 years, demonstrated at baseline a significant increase in BMS after the 2 h try out and had a minimum individual BMS improvement of 2 or more.

**Figure 5:**
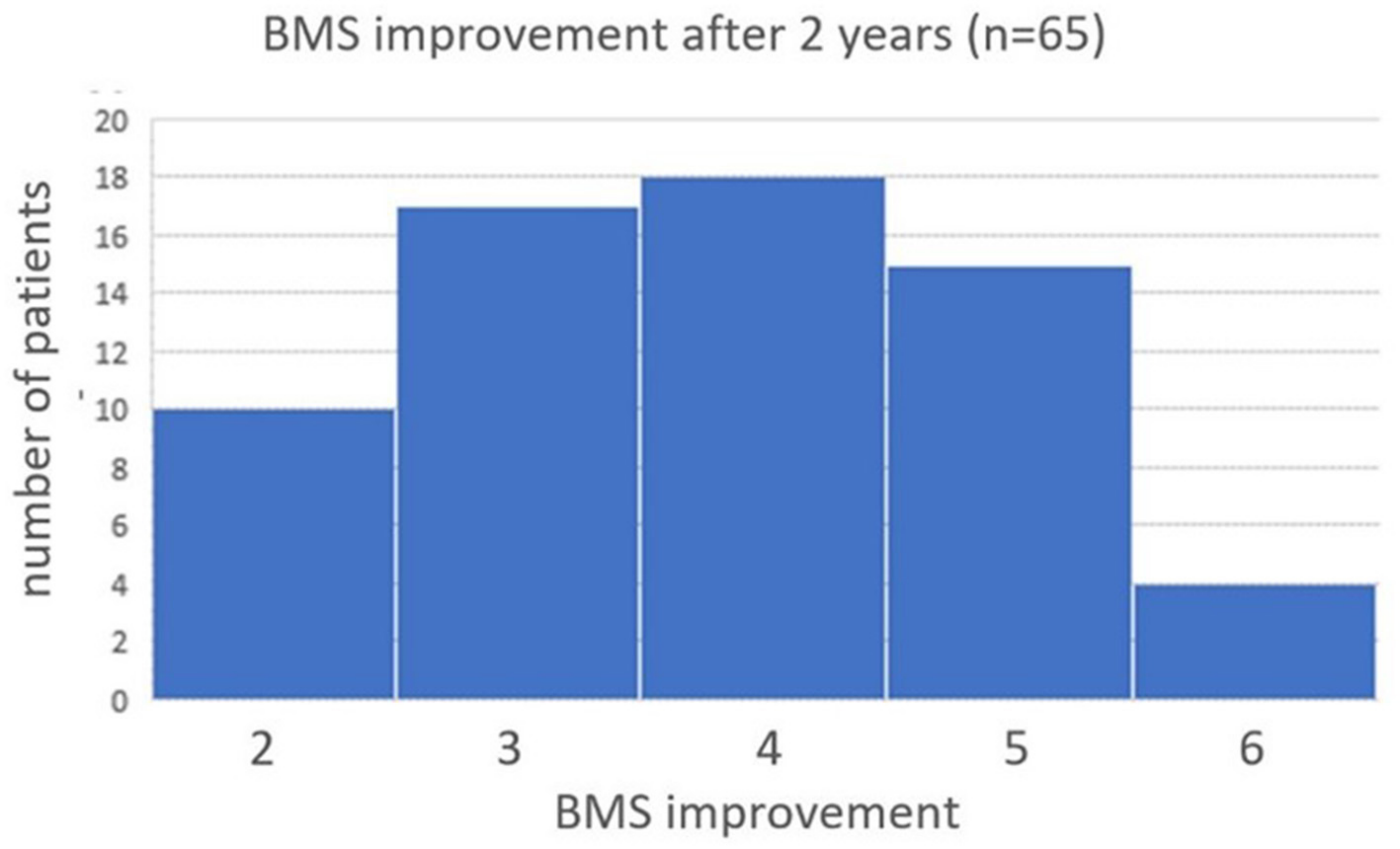
Histogram of BMS improvement after 2 years of Subconscious vibrotactile stimulation use. Frequency distribution of the change in BMS after 2 years of wearing the vibrotactile stimulation belt in daily life. The median improvement in BMS, compared to baseline, was 4 (range 2–6).

There was no more significant improvement in median BMS score after 2 months in the 65 patients who still wore the subconscious vibrotactile stimulation belt after 2 years. The improvement in median BMS by subconscious vibrotactile stimulation was stabilized after 2 months and was preserved over 2 years (Wilcoxon signed rank test, *p* > 0.05). All patients who could be contacted and remained in follow up between 2 weeks and 2 years, experienced a benefit of 2–6 points on the BMS scale compared to baseline without wearing the belt (Figure 5).

### Additional observations

Some additional observations were made during the follow-up consultations:

- All patients still using the vibrotactile stimulation belt after 2 months (*n* = 80), indicated that they had to wear the subconscious vibrotactile stimulation belt continuously during their activities, to benefit from the vibrotactile stimulation.
- Eleven patients reported that they started biking again.
- Four patients still using the subconscious vibrotactile stimulation belt after 1 year, asked for a smaller belt size because they lost weight. This might have resulted from increased physical activity.
- All patients wearing the subconscious vibrotactile stimulation belt for more than 1 year, mentioned that they could walk again without the need to pay attention all the time to balance. They were also able again to walk without a fear to fall, while turning and moving the head independently of the trunk.
- All patients wearing the subconscious vibrotactile stimulation belt for more than 1 year, felt more confident to go shopping again and merge in busy places, walk in the evening, resocialize, etc. Patients did not report any fall anymore.
- Seven patients wearing the subconscious vibrotactile stimulation belt for more than 2 years, restarted work again. They previously stopped working because of their BVP.
- All 2-weeks responders did not need any training: the additional vibratory stimulation pattern seemed to function as an intuitive additional information source to improve balance and gait.
- Four patients who decided to stop wearing the subconscious vibrotactile stimulation belt after 2 h of use, experienced the vibration as disturbing.

## Discussion

This study demonstrated that subconscious vibrotactile stimulation improves self-reported balance and mobility in a subgroup of BVP patients. The effect of subconscious vibrotactile stimulation becomes already clear after 2 weeks of daily use at home in many patients. After all, long-term adherence (1–2 years) is high in patients who experience an increase of 2 or more points on the BMS after 2 weeks of daily use.

Conform and in agreement with previous literature (20), balance and mobility can be improved by the vibrotactile stimulation in bilateral vestibular loss patients. It must be noted that a special subpopulation of bilateral vestibular loss patients was studied: only patients with very severe bilateral vestibular loss (VHIT, calorics, rotatory chair) were included. They all experienced a persisting reduced quality of life, reduced mobility, and poor balance (BMS ≤ 5), despite intensive vestibular rehabilitation and other treatments. On top of that, these patients were referred from all over the Netherlands because they were desperately searching for a treatment option. Therefore, all included patients were clearly motivated to regain quality of life and mobility.

The sensor in the balance belt is a 6DOF sensor but used to detect tilt and linear translations to provide the stimulation pattern. We hypothesize that the belt acts by increasing somatosensory substitution and not so much by replacing labyrinthine function as a tilt or translation sensor (otolith function). Further research including quantification of the otolith function might narrow down the inclusion criteria for the effectiveness of vibrotactile stimulation in severe vestibular loss.

The belt needs to be tightened on the waist to ensure proper somatosensory stimulation all around the waist. To achieve this was one of the most challenging design issues in the development of the belt over 20 years and made it clear that the belt needed to be elastic and that about seven different sizes are needed to optimally fit many humans.

**Figure 6:**
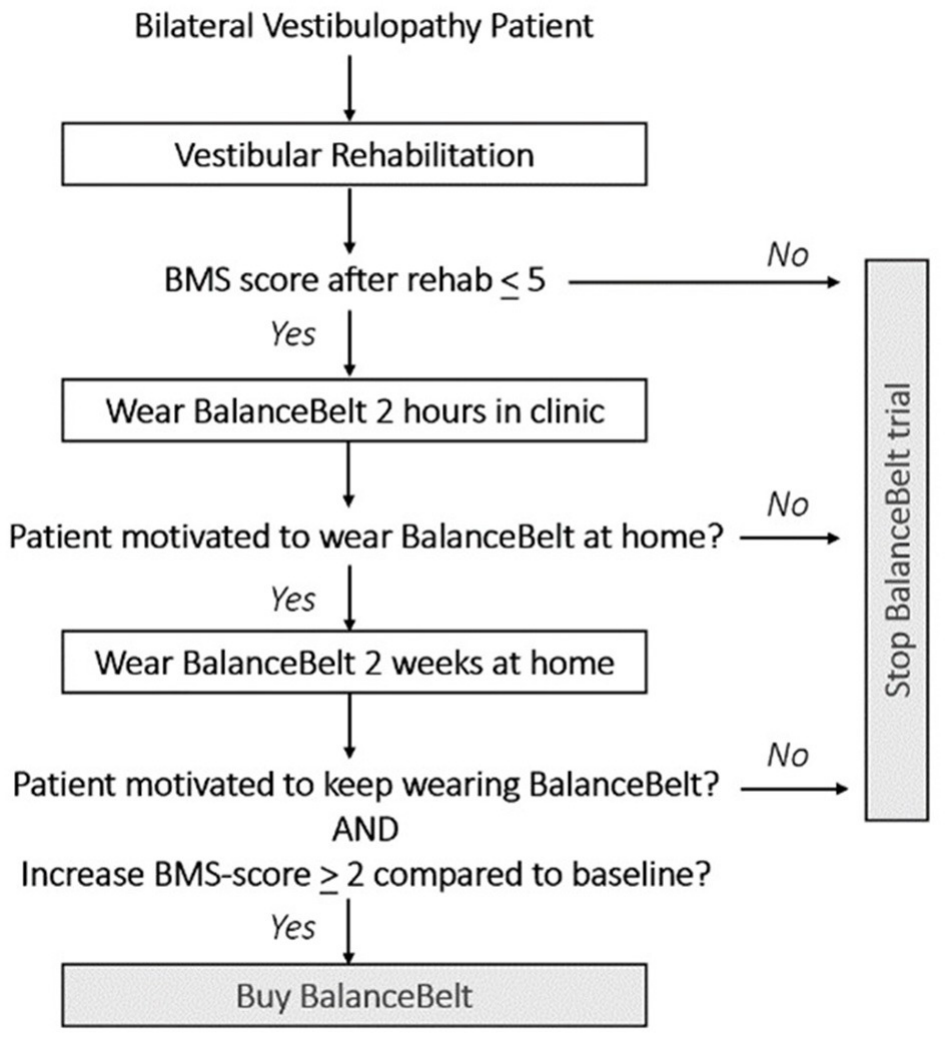
Proposal for an algorithm to select patients with bilateral vestibulopathy for the subconscious vibrotactile stimulation.

Management of vestibular hypofunction could be compared to the management of hearing loss. Regarding hearing, hearing aids, lipreading, sign language, or cochlear implants can be offered. Regarding vestibular hypofunction, vestibular rehabilitation, the subconscious vibrotactile stimulation belt or the vestibular implant might be possible options. However, the vibrotactile stimulation is currently the only available option when the traditional approach like vestibular rehabilitation is not effective. A vestibular implant is not yet clinically available (11, 21–33). Therefore, the subconscious vibrotactile stimulation belt might be offered as an additional treatment for BVP patients, in which vestibular rehabilitation was not sufficient. However, this study demonstrated that only a subgroup will benefit from it. It is therefore imperative to develop an efficient algorithm to select eligible patients. Since the effect of subconscious vibrotactile stimulation becomes clear within 2 h to 2 weeks for many patients, an algorithm taking these time frames into account, is proposed in Figure 6.

First, Bilateral Vestibulopathy Patients must have a low baseline BMS score ( ≤ 5). This cut-off was chosen based upon a pilot trial. It was previously found that patients with high baseline BMS scores, did not experience significant benefit from subconscious vibrotactile stimulation. Secondly, patients should try subconscious vibrotactile stimulation for 2 h in an outpatient setting. In this study, 26% of patients immediately indicated to experience no benefit from the belt after 2 h of use. This implies that in 26% of patients, the belt did not have to be taken home for 2 weeks. This decreases the amount of subconscious vibrotactile stimulations needed for testing. Thirdly, a follow-up consultation could be scheduled after 2 weeks, for a final decision about providing or buying the subconscious vibrotactile device. An increase of BMS score > 2 compared to baseline after 2 weeks of use predicted long-term adherence in this study. Surprisingly, an increase of BMS score > 2 compared to baseline after 2 h of use, also predicted long-term adherence in this study. However, still some potential responders only benefited after 2 weeks of use. Therefore, the authors hypothesize that a 2-week home trial is a more careful approach then to decide immediately after 2 h of use. A careful approach is desired, since subconscious vibrotactile stimulation is (still) relatively expensive. A conscientious selection algorithm might decrease costs for individual patients and society, comparable to hearing aid trial periods.

Proper patient instructions are crucial. Patients should be instructed not to pay attention to the vibration pattern, i.e., not to use the belt as a feedback system to control body posture. When patients are continuously paying attention to the belt, the body control becomes an additional cognitive task and cognitive load (37). Some patients are unable to “neglect” the vibration and experience the vibration and sound as disturbing. Additionally, an extreme fear to fall, anxiety and a long history of immobilization might make it difficult for patients to start using the belt. Proper motivation can be decisive, like with vestibular rehab, to reassure the patient to become more physically active again.

## Limitations

Three main limitations were identified with this study: (1) the BMS-score, which correlates well with the more general DHI in BVP (R-0.84), is a subjective balance and mobility score is a subjective score, (2) the adherence was reported by patients, not verified by e.g., accelerometers in the belt (20), and (3) this study was an open not blinded study, without a placebo control. However, it was previously shown in a group of responders, (20) that subconscious vibrotactile stimulation programmed in five different placebo modes did not significantly affect their BMS, Romberg (measuring sway area and sway velocity), gait, heel to toe walking, score on the Nintendo platform Ski-game, QOL score, Short Falls Efficacy Scale, or Dizziness Handicap Inventory. The same outcome parameters and the Timed Up and Go test, were also scheduled for assessment in the current study, but could not be evaluated due to the COVID-pandemic because patients were not allowed to enter the hospital. To further support the current evidence for the effectiveness of subconscious vibrotactile stimulation, a multicenter double-blind placebo controlled cross-over study is currently executed at Aalborg and Maastricht Universities. In this study, the impact of vibrotactile stimulation in BVP patients is quantified using multiple output parameters (BMS, Gait-analysis, Balance test, Timed up and Go test, and various questionnaires). Furthermore, the active use of the belt is measured by an actometer attached to the belt.

## Conclusion

Subconscious vibrotactile stimulation improves self-reported balance and mobility in a subgroup of BVP patients. Long-term adherence is high in BVP patients who experience an increase of two or more points on the Balance and Mobility Score after 2 weeks of daily use.

## Data Availability

The raw data supporting the conclusions of this article will be made available by the authors, without undue reservation.
